# Towards an understanding of GPs’ viewpoint on diagnosing postnatal depression in general practice: a small-scale realist evaluation

**DOI:** 10.1017/S1463423620000316

**Published:** 2020-10-09

**Authors:** Ashvanthi Nadira Sriranjan, Ruth Abrams, Geoff Wong, Sophie Park

**Affiliations:** Department of Primary Care & Population Health, Institute of Epidemiology & Public Health, UCL Medical School, London, UK

**Keywords:** diagnosis, general practice, GP perspective, postnatal depression, primary care, qualitative, realist evaluation

## Abstract

**Background:**

Less than half of postnatal depression cases are identified in routine clinical assessment. Guidelines and current literature suggest that general practitioners (GPs) may have an opportunistic role in detecting postnatal depression due to their early contact and existing rapport with many new mothers. There is limited research on the diagnostic approaches chosen by GPs in different GP−patient contexts. Our small-scale study evaluates the thought processes of seven GPs based in one practice when forming a clinical diagnosis of postnatal depression under different contexts.

**Methods:**

Seven GP participants were interviewed using case vignettes about postnatal depression, based on an adapted Johari’s window framework. A realist approach to analysis was undertaken with the intention of understanding GPs’ responses to different situations. Context−mechanism−outcome configurations were constructed, and a programme theory was formed to consolidate the findings.

**Findings:**

Findings suggest that diagnoses may be a clinician-led or collaborative process between GP and patient. In collaborative contexts, stigmatising views were addressed by GPs, time for self-reflection was encouraged and mothers’ views were accounted for. Clinician-led diagnoses often occurred in contexts where there was a lack of acknowledgement of symptoms on behalf of the patient or where safety was a concern. The personal and clinical experience of GPs themselves, as well as effective communication channels with other primary care professionals, was significant mechanisms.

**Conclusion:**

GPs use a variety of strategies to support patient disclosure and acceptance of their condition. The complexity of GP−patient contexts may influence the clinical thought process. We address some of the gaps in existing literature by exploring postnatal depression diagnosis in primary care and provide tentative explanations to suggest what works, for whom and in what contexts.

## Introduction

Postnatal depression is a mood disorder and mental health condition that affects approximately 10−15% of postnatal women (RCPSYCH, 2018). It is an important public health problem because of the severe long-term impacts on maternal mood, the mother−baby relationship, and the baby’s emotional and cognitive development (Department of Health, 1999; MIND, 2016). However, less than 50% of cases are identified in routine clinical assessment, making postnatal depression a challenging issue to address (Paulden *et al.*, 2009). Prevention and early detection is becoming increasingly significant in mental disorders and has made headline news with Ron Kessler, a Harvard Medical School professor, stating that current approaches in psychiatry are like, ‘practising 1950s cardiology, where you wait for a heart attack and once it happens you know what to do’ (Rice-Oxley, 2019). Yet underdiagnosis remains a problem. This may be influenced by factors such as self-awareness, the condition’s stigma and societal pressure on new mothers to feel confident in their maternal role (RCPSYCH, 2018).

General practitioners (GPs) are often involved in regular postnatal check-ups placing them in a unique position to monitor mothers’ well-being (National Health Service (NHS) Choices, 2017). They are sometimes the first point of contact for many mothers during pregnancy and postpartum fostering a longitudinal relationship with their patients (Earls, 2010; Neiman *et al.*, 2010). They are therefore in a good position to recognise postnatal depression and be able to identify, treat and where needed triage severe cases to secondary care (Department of Health, 1999). Current National Institute for Health and Care Excellence (NICE) guidance suggests that during a consultation with a new mother, GPs should consider asking whether they have (1) experienced feelings of low mood, depression or hopelessness and (2) noticed a change in their behaviour such as having little interest in doing things (Whooley *et al.*, 1997; NICE, 2017). If either of these questions are answered positively, guidance suggests that a GP should consider using a screening tool such as the Edinburgh Post-natal Depression Score or Patient Health Questionnaire-9. NICE also recommends exploring any personal or family history of mental illness. This should alert GPs to both significant risk factors and possible symptoms of postpartum psychosis (NICE, 2017).

A GP’s clinical judgement therefore plays a significant part in a patient’s assessment which may be influenced by a range of different factors, such as risk tolerance and experience (Kienle and Kiane, 2011). Exploring what factors are at play during a consultation may go some way to understanding the underdiagnosis of postnatal mood disorders in postnatal women. Whilst literature crucially acknowledges that the causes of underdiagnosis are complex, we continue to have an incomplete understanding (Hayes, 2010; Neiman *et al.*, 2010; Gjerdingen *et al.*, 2011). Within the existing literature identified, there is limited exploration of what the contextual influences on GPs’ chosen diagnostic approaches are. For example, in terms of examining the context in which the diagnosis is developed, Celik *et al.*’s (2016) study identified patient contexts that could predispose them to postnatal depression, and Mishina and Takayama’s (2009) study explored how effective screening tools are in various population contexts. However, neither of these studies investigated the influence of doctor−patient context on the diagnostic process nor used the process of realist logic to analyse how diagnoses are made. Realist logic is based on the concept that different people respond in a variety of ways to the same experiences based on the personal, social, historical and cultural contexts of the setting in which they are in (Wong *et al.*, 2013). Understanding how these contextual factors might alter the execution of the postnatal depression diagnostic process could help address the existing research gap and contribute to improving diagnostic policies, so that they are more applicable to individual circumstances.

## Study aim

Our small-scale study explores the perspectives of seven GPs based in one practice regarding the diagnosis of postnatal depression and uses a realist approach to analyse findings. Understanding the GP’s perspective may provide insight into how guidance might be adapted to suit the different contexts arising during the screening of a new mother’s well-being. Therefore, we frame our study in order to explore the following:

1. How does the GP−patient context influence the actions taken by GPs when diagnosing postnatal depression?
2. How do GPs’ postnatal depression diagnostic approaches in different contexts influence the desired clinical outcomes in postnatal mental health consultations?

## Methods

Our intention was to gain an understanding of the thought processes GPs take when negotiating the boundaries between stressful life experience and a clinical diagnosis of postnatal depression (Epifanio *et al.*, 2015). To account for the relationship between the context, diagnostic approach and outcome in this study, a realist analytical approach is used. A realist approach can be particularly useful for researchers trying to establish what works, for whom, and in what contexts (Rycroft-Malone *et al.*, 2012). It enables complex topics, such as making a postnatal depression diagnosis, to be analysed so that causal processes can be explored and formed.

**Figure 1.**
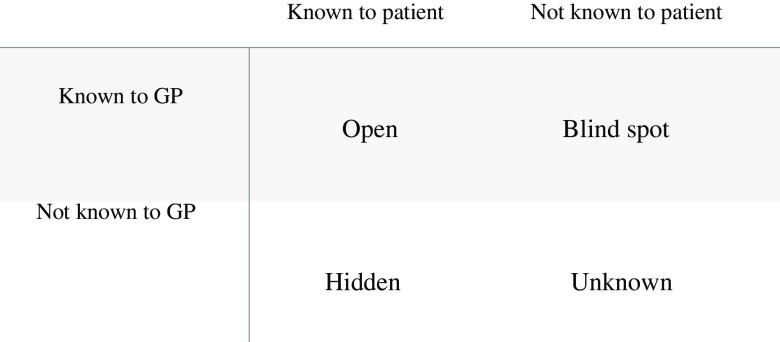
Initial programme theory: Johari’s window adapted for the GP−patient interaction

Unlike many physical illnesses, the diagnosis of postnatal depression is not straightforward. For instance, there is no blood test that can confirm or rule out the diagnosis like there is for diabetes or a thyroid disorder. In order to situate this study, we are using patient disclosure as our starting point. We conceptualise the diagnostic challenge in this way because existing literature suggests that patients speaking honestly and having insight into their emotions is a highly complex and necessary aspect of the diagnostic process (Earls, 2010; Hayes, 2010; Neiman *et al.*, 2010; Gjerdingen *et al.*, 2011). If a patient does not disclose his/her feelings and the GP is not aware, then no diagnosis will be reached. If a patient does not disclose but the GP suspects, then they may still make a diagnosis and share their concern with the patient. Thus, the insight of both the patient and the GP is crucial in encouraging the diagnosis to progress. However, certain circumstances impose barriers for patients to speak openly about their emotions. To help us understand the ways in which patient disclosure may arise, we have applied Johari’s window (Luft, 1969). Johari’s window is a two-dimensional framework used to identify an individual’s known and unknown needs in the context of learning and self-reflection (Luft 1969; Hutt *et al.*, 2015). Given the importance of GP−patient interactions and disclosure in this process, we framed our initial programme theory around Johari’s window and the idea of knowns and unknowns. Using Johari’s window as our initial programme theory enabled us to envisage four diagnostic situations: (1) open (the patients’ emotions and how they are coping are known to both GP and patient), (2) blind spot (postnatal depression recognised by GP but not patient); (3) hidden (postnatal depression known to patient but not GP) and (4) unknown (postnatal depression unknown by both patient and GP). We were then able to refine this programme theory, through our data analysis to produce additional insights and develop our final programme theory (Figure 1).

## Ethical approval

Ethical approval was obtained from the University College of London and the Health Research Authority before data collection began. This study was registered following university ethics procedures. All interviews were confidential and anonymised by the first author. All audio recordings were deleted once transcribed, and all transcripts and consent forms were stored in a password-protected computer.

## Data collection

We used semi-structured interviews with GP participants in North London to collect the data. We began our interviews by asking participants about their professional background. We then presented a set of patient cases which were devised to stimulate participant discussion about identification and management of postnatal depression in a range of situations. These case vignettes were developed by applying a Johari’s window framework to the clinical encounter interview vignettes. Different clinical presentations of mental illness were used to create cases representing each of the Johari’s window quadrants (Text Box 1 in the Supplementary Material): Case 1 refers to the ‘Unknown’ diagnostic situation of Johari’s window using the concept of somatisation where physical symptoms are the presenting complaint of a mental illness (Busaidi, 2010) which reduces GP and patient awareness. Case 2 refers to the ‘Hidden’ scenario by limiting what the patient expresses to the GP. Case 3 refers to the ‘Blind Spot’ scenario as the patient has limited self-insight but is still presenting with symptoms. Case 4 refers to the ‘open’ scenario as the patient presents with the diagnosis herself. The cases were initially created by A.N.S. and then developed further with the support of S.P. who is a practising GP to improve their clinical accuracy and suitability for our research question. We also asked general questions on postnatal depression diagnosis such as ‘why do you think postnatal depression may be underdiagnosed?’ and ‘what are your views on the use of screening tools to diagnose postnatal depression?’ (Appendix 1 in the Supplementary Material). A.N.S. conducted the interviews, one to one during March and April 2018 in the practice GPs worked at to maximise participant convenience. Interviews lasted between 40 and 60 min and were audio-recorded and transcribed verbatim (by A.N.S.).

## Recruitment and sampling

Our initial recruitment request went to seven practices within North London by emailing their respective practice managers. We received a positive response from three practices; however, GPs were only recruited from one practice due to limited capacity with the two other practices. We made contact with GPs who had expressed an interest through the GP partner who acted as a gatekeeper. We used convenience sampling so that any participant who showed interest could be recruited. Overall, seven GPs were recruited. Two participants were trainees with less than 3 years’ experience. The remaining five participants had a range of 6−37 years’ experience in general practice. Two participants conduct postnatal check-ups frequently. Four participants were female and three were male. A breakdown of participant characteristics can be found below (see Table S1 in the Supplementary Material).

## Researcher reflexivity

A.N.S. kept a reflective journal during the interview process to record ongoing reflections. This was used during analysis, for example, participants’ non-verbal behaviour, connections between data and literature and areas for future enquiry. Iterative changes to the study design were also recorded in the journal. Reflections from this journal were discussed with R.A. and S.P. during data analysis to aid sense-making processes and clarification (Finlay, 2002; Ortlipp, 2008).

As a medical student, A.N.S. has some knowledge on the clinical aspects of mental health and has observed GP−patient consultations where diagnoses have been developed and established. This may have influenced the content of the case scenarios (see Text Box 1 in the Supplementary Material) as well as how interview data were conceptualised into a final programme theory. Therefore, the programme theory was reviewed by the other authors to reduce this bias and improve how accurately the theory reflects the data collected. R.A. has no clinical experience but has experience in primary care workforce research which may have helped to shape our overall interpretation of how GPs and other primary care professionals work. Both G.W. and S.P. are practising GPs with experience in diagnosing mental illnesses. Therefore, they may have influenced how we portrayed what GPs’ views on postnatal depression diagnosis are in specific situations, especially if a context−mechanism−outcome configuration (CMOC) relates to a personal experience they have had.

## Data analysis

A number of factors may contribute to a GP’s awareness of postnatal depression. For example, personal experience, good lines of communication with other health professionals and/or the ability to spot subtle cues may all aid in a diagnosis. Our programme theory therefore developed from an initial use of Johari’s window to conceptualise the diagnosis as a process of known and unknowns by patient and GP. The analysis of the interview data added a deeper understanding of the conditionality of this process to the programme theory, in particular, the relational aspect of this interaction and how this shaped the possibility for patient and clinician insight and diagnostic acceptance. We used the framework advocated by Wong *et al.* (2013), Pawson and Tilley (2004) and Papoutsi *et al*. (2017) to analyse our data. This framework allowed us to examine GPs’ explanatory models for diagnostic decision-making of postnatal depression. This analytical framework included (1) familiarisation with the data, (2) coding the data, (3) conceptualising the patient pathway for diagnosis, (4) development of context−mechanism−outcomes and (5) reviewing context−mechanism−outcomes and the patient pathway. We began analysis in parallel with interviews, so that we could move iteratively between data, the interview topic guide and our programme theory. Recurrent patterns within the data were identified and summarised and then developed into CMOCs (Pawson and Tilley, 2004) (Appendix 2). During ‘data clinics’, interview data were discussed with R.A. and S.P. in relation to the research question and to compare interpretations, so that the final CMOCs and programme theory could be developed.

## Results

The following section outlines the CMOCs supported by the interview findings. To build explanations of how GPs come to a diagnosis of postnatal depression, we decided to focus on what GPs do in situations that may involve more uncertainty. Encouraging an honest dialogue with patients can be a challenging interaction (Hayes, 2010), so we were particularly interested in how GPs discussed this experience. We were also interested in how GPs valued the role of screening tools (Mishina and Takayama, 2009; Hayes, 2010; Neiman *et al.*, 2010).

We used case vignettes as the basis for discussion about approaches to patient diagnoses. Our findings suggest that a diagnosis may happen in one of three ways: (a) through patient−GP co-construction (by which we mean a collaborative effort between patient and GP (Vickers *et al.*, 2012), (b) as a clinician-led diagnosis or (c) as a result of GP awareness. These ways are discussed in more detail below along with underpinning CMOCs and illustrative examples of the data we drew on.

### Co-construction of a diagnosis

Co-construction of postnatal depression diagnosis may require a GP to break down a new mother’s misconceptions about their feelings, hold space for patient self-reflection and wait for a new mother to offer up their own thoughts.

#### CMOC 1: When GPs normalise low mood in the postnatal period (C), mothers are more likely to disclose they have such a problem (O) because they feel less stigmatised (M)

Some GPs discussed how the experiences of being a new mother, particularly in the early stages of being postnatal, can sometimes give rise to unexpected feelings. These feelings may clash with anticipated and expected emotions and so when not felt, may create a fear of judgement in new mothers. In this context, we identified that some GPs felt their role to be one of the educating mothers on how common and acceptable unexpected feelings can be amongst new mothers (e.g., overwhelmed, low mood).

‘*Mums don’t expect to feel depressed after they’re pregnant because many think it’s going to be an exciting time. If they are depressed, it kind of rocks the boat with them a bit and they feel like they’re in the minority. It’s important that we tell them that it’s okay, and you’re not alone, or they- they won’t tell us these things*’. (Alisha, GP trainee, 3 years’ experience)

For GPs who felt their role to be one of education, they would shape the conversation around normalising feelings of low mood as a way to encourage self-reflection within their patients. For GPs in this position, patient disclosure relied on breaking down associations between how a new mother thought they should be feeling compared to what they were actually experiencing. GPs discussed encouraging mothers to try and speak freely about their emotions. Holding space in this respect meant that a patient may subsequently feel safe enough to share their emotions, open up and be receptive to an appropriate diagnosis.

‘*I would often say [to the new mum] that having a baby is really difficult and sometimes people do suffer from feeling very low after they’ve had a baby and that’s not unusual, has that happened to you? I would kind of normalise it…that’s kind of a good way of getting someone to admit…the minute you say is it happening they just burst into tears and open up*’. (Rachel, 6 years’ experience)

Some GPs felt that discussing depression beyond its stereotypical symptoms may support patients in their own identification or willingness to explore a potential diagnosis.

‘*I mean often I say to patients people can suffer with depression and not with your typical crying all the time and being sad…you can have all sorts of things and physical symptoms and try and go down that route…sometimes people don’t want to admit that there’s something more in their head rather than something physical, but I think if you tell them…then they’re more willing to explore that’*. (Ajay, 8 years’ experience)

Reducing stigma by effective holding, the space for new mothers to explore their feelings could help normalise a range of emotions felt by new mothers. Giving mothers this emotional outlet and educating them could help to reduce the impact of stigma surrounding mental health issues. This may facilitate patient disclosure and consequently a co-construction of a diagnosis.

#### CMOC 2: When patients are prepared to disclose a mood disorder (C), GPs feel they have ‘permission’ (M) and so explore the condition with patients collaboratively (O)

From a GP’s perspective, being involved in a patient’s journey to their own self-diagnosis may be extremely beneficial. For some participants of this study, having the patient recognise symptoms of their own volition may facilitate a more collaborative effort in terms of taking steps towards recovery.

‘*So I think when people feel low and recognise that, that’s really positive and a good thing- you’ve seen it, you’ve spotted it and you’ve come for some help- well done you. We can now work on this together*’. (Barry, 25 years’ experience)

For some participants of this study, patient insight or self-reflection was perceived as a ‘gift’, something that a patient actively decides to ‘give’ to the GP as a way of asking for help.

‘*No one can actually get inside your head, if you do feel that you’re depressed then you’re more than likely depressed. So it doesn’t matter what I think because as soon as someone says they think they might be depressed that suggests mental health issues. What’s incredible here is that getting to the diagnosis is difficult, but if she’s given it you, then you can understand what’s going on together because you need her insight into it all to get there*’. (Vincent, 5 years’ experience)

Some participants of this study appeared to view patient insight as being highly valuable to the diagnostic process because of its ability to aid in the knowledge sharing of symptoms and consequently the construction of a more detailed and accurate diagnosis.

### Clinician-led construction of diagnosis

#### CMOC 3: When a mother is reluctant to accept they have a mood disorder (C), GPs will use a screening tool (O) because it legitimises their diagnosis (M)

Conversely, a clinician-led diagnosis may present in instances of high risk or when a patient requires a degree of prompting in relation to their symptoms. In this instance, screening tools may support a GP to manage patient risk or educate patients on their mental health levels. Some GPs discussed how screening tools could be beneficial in aiding a diagnosis of postnatal depression. GPs discussed how, in some cases, screening tools may legitimise the diagnosis of postnatal depression in the same way that blood tests legitimise the diagnosis of diabetes. The tools could help patients consider the possibility that their mental health levels are lower than they perceived them to be.

‘*It’s like showing them a test result, like if you told someone you have diabetes because look your blood sugar levels are high. It validates what you’re saying when they don’t accept it and I think that’s quite useful*’. (Barry, 25 years’ experience)

A screening tool may help a clinician in planting a seed in a patient’s mind about what might be wrong with them. In this respect, a screening tool may act as a mirror, reflecting a patient’s symptoms back to them and affording a GP a degree of control in terms of managing next steps.

‘*But sometimes it is a case that it’s useful to help reflect back to someone and say okay these are the features that suggest you may have depression- and actually it’s interesting, you’ve ticked all of them, what do you think about that? Sometimes seeing it in black and white just helps someone to- to realise and to understand….So I would actually only do it if I felt that this person actually needs some persuading of what the problem is*’. (Rachel, 6 years’ experience)

#### CMOC 4: When a mother is reluctant to accept she has a mood disorder (C), GPs will organise a follow-up visit (O) because they think that the patient needs time to reflect (M)

Another way in which GPs would facilitate a diagnosis was by encouraging a follow-up visit. For example, when patients were reluctant to engage in a mental health discussion, some GPs explained that booking a follow-up visit may help build a degree of familiarity and continuity with a patient. Some GPs felt that this may also provide a patient with some time to explore emotions independently after the initial discussion.

‘*What I would probably do is kind of bring her back- kind of plant the seed there and then say okay let’s see how things go. I’d definitely want to monitor her whilst she does some self-reflection and- until she’s ready to come in and talk to me about it really and what she’s realised over that time*’. (Rachel, 6 years’ experience)

#### CMOC 5: When a mother is a risk to herself or others (C), GPs will act as needed (O) because they feel it is the safest thing to do (M)

Patient safety and a GP’s tolerance of risk were also significant in their clinical approach. For example, some participants expressed the importance of assessing the safety risk a patient poses on themselves and their baby. High-risk scenarios would inevitably require a clinician-led diagnosis.

‘*Yeah, I mean sometimes I might take the lead- if for example I think they might be in denial then I would take the lead. You wouldn’t Section [a form of compulsory mental health assessment] them if you didn’t have to- you’d try and get patients to join in on making the diagnosis. If they’re psychotic or suicidal or the baby’s in danger, then I would need to get to that diagnosis faster and admit them and get them seen by a crisis team’.* (Fiona, 37 years’ experience)

Unlike contexts in which a GP is able to use clinical judgement and conclude that a patient might be low risk, patients in immediate danger tend to be managed much more quickly, with less time to facilitate patient reflection and dialogue.

### Development of GP’s awareness

#### CMOC 6: When GPs see new mothers (C), they are more likely to make a diagnosis of postnatal depression (O) if they have a high index of suspicion (M)

Some GPs suggested that a reason postnatal depression is underdiagnosed is because GPs miss the subtle cues that may be masked either by paying attention to the wrong things, undertaking physical examinations that detract from a diagnosis or being unfamiliar with the condition.

‘*We’re not very good at asking about it, and patients are not very good at telling us about it. Uhm, we can get very coo-ey about little babies and miss very important things which I guess comes from experience. So every time you uhm, you see a new mother you need to keep it as a potential at the back of your mind or they won’t tell you’.* (Vincent, 5 years’ experience)

One GP felt that having a personal experience (e.g., a close friend with postnatal depression) may increase clinical awareness as it improves insight into the real-life difficulties of postnatal depression and the importance of early detection.

‘*Since then, I’ve probably been- it’s very much on my radar because I don’t want anyone to go through this ever again. (Yeah.) I want to feel like I’m doing my job in terms of looking out for these women and want them to share these things with me, so I think that anything that happens to you personally is going to massively change your perspective on things*’. (Rachel, 6 years’ experience)

#### CMOC 7: GPs are more likely to make a diagnosis of postnatal depression (O), when they are alerted by others (C) because it raises their index of suspicion (M)

Other GPs discussed how their awareness of a patient’s condition may come from unexpected knowledge sources such as receptionists, who may greet a new mother and see their stress or anxiety first-hand.

‘*You know people come in and see them and sometimes the receptionists see them and if the mums are stressed and they cry then the receptionists can bring that to our attention. The receptions see patients first most of the time so we’re constantly trying to train them to be on the look out for all that stuff*’. (Barry, 25 years’ experience)

Some participants discussed how this communication channel also exists with health visitors who do home visits. Health visitors are registered nurses or midwives with additional training in public health enabling them to effectively assess the health needs of individuals, families and the wider community. In the context of postnatal depression, health visitors assess the family and home situation of mothers, can help identify mothers at risk of mental illness through the use of screening tools and their clinical insight, and refer them to receive further support (Peckover and Aston, 2018). This is important as a patient’s apparent health at the surgery may differ from the reality of their health at home. Olander *et al.*’s (2019) paper expresses how home visits are one of the ‘triad of core practices’ as they form an opportunistic setting for building rapport and uncovering withheld health needs.

‘*That’s why health visitors and midwives do initial assessments at home because you see- you get an idea of how someone’s coping. So I think that- sometimes when you see frail elderly people they seem fine but then you go and visit them and their house is in an absolute state, there’s no fresh milk in the fridge and you think oh my goodness’.* (Rachel, 6 years’ experience)

Lines of communication with other health professionals and the degree to which information is fed back may go some way in helping to diagnose a new mother with postnatal depression because of the clues that are picked up during this home visit. However, a few participants described how government policy directives have changed the structure of working for health visitors and the impact this has had on interdisciplinary communication and continuity of primary care services for mothers (Derrett and Burke, 2006; Olander *et al.*, 2019). For example, health visitors are no longer allocated to individual practices and instead work collectively and are assigned to wherever there is patient demand with the intention of increasing shared workload and professional support (Cowley *et al.*, 2013; Aquino *et al.*, 2016).

‘*We used to have health visitors who were allocated only to the practice and ermm, now they work across areas. So now we have a million different health visitors and there’s no communication or less communication which makes everything hard because we don’t get the information we need*’. (Fiona, 37 years’ experience)

**Figure 2.**
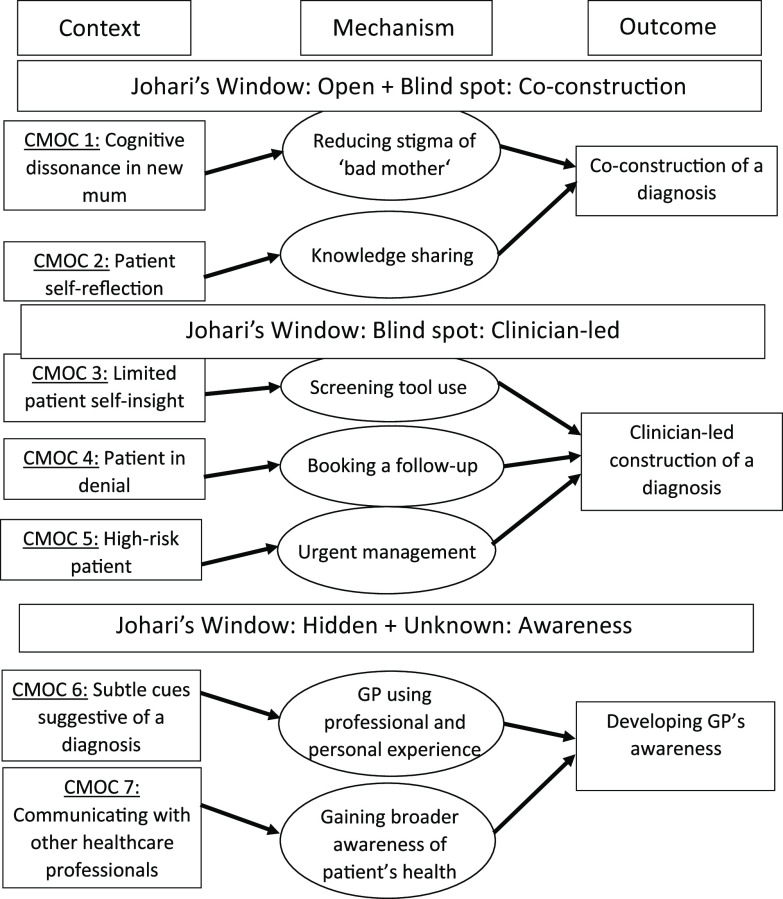
Final programme theory

Some participants expressed how this change in a health visitor’s structure of working has reduced the communication channels between individual GPs and health visitors. This may have limited the ability of GPs to gain a broader awareness of a patient’s health levels (Figure 2).

## Discussion

### Summary of findings

Our small-scale realist evaluation explored the thought processes GPs take when considering the diagnosis of postnatal depression. We aimed to understand how clinical decisions may be modified according to GP−patient context using a realist analytical approach. Consequently, our CMOCs focus on the challenges of an honest dialogue between GP and patient, especially in cases of clinician uncertainty and patient stigma. We identified how the diagnosis can either be made as a clinician-led diagnosis or as a co-construction by the GP and patient working together. Whilst co-construction of the diagnosis may aid rapport, a clinician-led diagnosis may be beneficial if patients show a lack of self-insight, are in denial or are high risk. The ability of a GP to detect subtle cues was also found to be important in improving their clinical insight. This awareness was shown to be affected by factors including GPs’ personal and professional experience and their communication channels with other health professionals. The role of screening tools suggests that they provide more use for clinicians to help legitimise the diagnosis for the patient rather than helping the clinician form the diagnosis itself. Overall, our study provides emerging insight into how GPs address some uncertainties they face when developing a diagnosis of postnatal depression. However, due to us only interviewing seven GPs, these CMOCs should only be considered as preliminary. Additionally, as all participants were based at the same practice, this further reduces the variation of data collected as the GP participants were likely to discuss cases and align how they practice. A key purpose of our realist approach is to show that findings may be transferred to different contexts but may also be disproven. Further research with larger participant sample sizes including a greater number of practices would help confirm, refine and refute CMOCs we have interpreted, help us understand further CMOCs that we did not identify and hence more accurately reflect views of the GP population on postnatal depression diagnosis.

### Comparison with existing literature

#### Co-constructing a diagnosis of postnatal depression

One significant area this study explored was the co-construction of a diagnosis of postnatal depression. A way in which GPs attempted to provide an environment for co-construction was reflected in their attempts to educate mothers on how common these difficulties are. Normalising these feelings so that patients see there is no shame in having them may encourage patient disclosure. Increasing mothers’ awareness of postnatal depression may involve its diverse presentations, such as the concept of somatisation. This could promote self-reflection of their emotions further. Addressing a patient’s misunderstandings as well as any fears of judgement may go some way in reducing stigma (Bursztajin and Barsky, 1985). GPs, who identify a patient’s existing views, offer a rationale and check their understanding may also support an effective consultation structure between a new mother and GP (Cohen-Cole and Bird, 1991).

A self-diagnosis of postnatal depression was also shown to support a co-construction of the diagnosis. Participants of our study expressed how bringing out self-awareness is the aim for many mental health consultations. Increased patient awareness enables patients to become an active participant in constructing their diagnosis and may help in developing a more detailed and accurate diagnosis. This insight is an important component for shared decision-making and treatment compliancy in the psychiatric context (Veatch and Guidry-Grimes, 2019). Understanding the nature of their condition may help patients understand the importance of receiving support and increase how engaged they are in their personal healthcare plan. For example, this may be encouraged by asking patients to contribute their own ideas, concerns and expectations during a consultation (Kurtz and Silverman, 1996). Our findings reflect this concept of patient insight being a ‘gift’ to the consultation, and a self-diagnosis of postnatal depression may enable both the patient and GP to have input into the patient’s healthcare.

### Directing the diagnosis of postnatal depression

A clinician-led diagnostic approach appeared necessary if some mothers were unwilling to consider the idea of a mental illness due to limited self-insight or being in denial of issues they are facing. In these contexts, screening tools may aid in a diagnosis by providing an objective perspective for the patients. As the tool can act as an impartial view of the patient’s symptoms, it could help encourage acceptance in the same way a blood test legitimises the diagnosis of diabetes. Hence, our study looks at how screening tools can benefit patient awareness rather than formulation of the diagnosis itself. Existing literature on screening tools generally emphasises their accuracy in forming a diagnosis of postnatal depression and hence their potential benefit for the clinician’s awareness (Mishina and Takayama, 2009; Hayes, 2010; Gjerdingen *et al.*, 2011). Our study however identified an alternative purpose for screening tools in legitimising a diagnosis for a patient to encourage patient acceptance of their condition.

Some participants explained that patients who are reluctant to discuss mental health may benefit from follow-up visits. This could give the patient time to increase their familiarity with their GP and reflect on their feelings and emotions. Continuity between GP and patient can help them view the primary care setting as a safe, non-judgemental environment and view their GP as someone who values their thoughts and feelings (Earls, 2010; Neiman *et al.*, 2010). Patients may also be more likely to share their vulnerable feelings with a GP they have built a rapport with which inevitably only develops when a consultation affords both the patient and GP time (Pendleton *et al.*, 1984).

Clinician-led construction of a diagnosis appeared to also be significant when considering the temporal necessity of a diagnosis. For example, if a mother appeared to be at risk of harming herself or her baby, then a paternalistic diagnostic approach may be essential (BMJ Best Practice, 2019). Our findings align with existing literature that suggests those at high risk of suicide require a plan of immediate intent as safety maintenance is the highest priority (Orsolini *et al.*, 2016).

### The role of a GP

Understanding the condition of a mother’s mental health and knowing the most appropriate clinical approach to take with her are important insights for diagnoses to be reached effectively and as early as possible. One factor affecting this level of insight appeared to be a GPs’ receptiveness to patient cues. Navigating the dynamics of a consultation whereby patients may not provide their symptoms openly but may instead present ‘clues’ through both their speech and behaviour was sometimes a way in which GPs were able to determine a diagnosis. Many GPs depend on clinical intuition when considering the possibility of a postnatal depression diagnosis (Chew-Graham *et al.*, 2008). However, the ability to detect these clues may be affected by the GP’s level of experience (Yawn *et al.*, 2012). Whilst training on postnatal depression screening and diagnosis for GPs may correlate with earlier diagnoses and improved clinical outcomes (Yawn *et al.*, 2012), personal experience may also increase GPs’ awareness of the real-life difficulties imposed by postnatal mental illnesses (Salinsky, 2003).

Although GPs primarily develop their clinical awareness by interacting with their patients, communicating with the primary care team may also be beneficial. For instance, receptionists are the first to see patients’ general appearance. Community midwives coordinate a mother’s pregnancy journey and provide continuity of care. Health visitors are privy to how mothers are and how the mother−baby relationship is at home. This makes observing and assessing maternal mental health a key opportunistic aspect of their roles. Hence, these additional but often minimised communication channels from other primary healthcare professionals may provide a unique clinical insight for GPs. Although many primary care clinicians view themselves as the leader in the healthcare team and so may collaborate less with other professionals, models of care that utilise interprofessional collaboration for diagnoses have shown to improve patient safety and lead to better clinical outcomes (Hodgson *et al.*, 2017).

## Limitations and future research

This was an exploratory evaluation, which drew on a small convenience sample of seven GPs based in one practice and used only one method for data collection (interviews). However, within these limitations, the data were collected and analysed in a rigorous manner. In addition, our findings have been presented transparently, thus enabling readers to clearly appreciate what data we interpreted to build our CMOCs. To further strengthen the explanatory powers of our CMOCs, we have deliberately sought analogy of our findings with existing literature. In other words, we made attempts to check how well (or not) our findings fitted in with relevant pre-existing literature (Wong *et al*., 2018). Despite these, further empirical data are likely to enhance the explanatory powers of our CMOCs and programme theory. Future studies may wish to collect more data from a wider range of GPs and introduce a means of checking if self-reported clinical practice is actually carried out as reported. Postnatal depression diagnoses are not a universal process, what works for one GP−patient consultation may not work for another. With future research building on ours and helping identify further CMOCs, we could contribute to policies becoming more adaptable to the context of a GP’s level of awareness and patient’s level of insight. Using CMOCs to find effective methods to reduce stigma and educate patients and GPs on the signs of postnatal depression in different situations could improve early diagnostic rates and hopefully outcomes by reducing the number of cases that progress to a severe state.

We focused exclusively on GPs in order to understand their perspectives. We did not explore the diagnosis making process from the patients’ perspective. This would have been valuable and could be the focus of future studies. Hearing from patients who have been diagnosed and whether the method of diagnosis was effective in relation to the contextual factors they were experiencing may be useful in helping bring further clarity to different CMOCs. Another group we did not gather data from was other health professionals, such as health visitors, receptionists and midwives. Their experiences in the management of women with postnatal depression might contribute additional valuable data to help us better understand the diagnostic processes for GPs. Community midwives provide continuity for women through pregnancy and postnatally to ensure they receive effective support and care. Receptionists are the first primary care form of contact many of these mothers have and so may be able to detect clues that GPs miss. Interviewing health visitors may have been beneficial in helping us further appreciate the nature of professional−patient contexts that influence screening of mental illness and how that screening process is carried out. Furthermore, it could have helped us gain a deeper understanding of the communication channel between GP and health visitor regarding maternal mental health.

## Conclusions

The complex nature of GP−patient interpersonal interactions during a postnatal depression diagnosis may shape the clinical approach chosen and the resulting outcome. Patients presenting as aware of their condition may be more willing to seek collaborative solutions to improving their mental health. In instances where patients are unaware, screening tools present as a useful strategy used by GPs to support patient acceptance of their condition. The subtlety of cues either noticed by the GP or provided to them by other staff members may also contribute to an effective postnatal depression diagnosis. Taking a realist approach to exploring postnatal depression and its diagnosis has enabled an exploration of these contexts, mechanisms and outcomes. We propose tentative configurations to suggest what works, for whom and in what contexts. Implications of this include acknowledgement of the different points at which a patient and GP ‘meet’ psychologically and the influence this has on effective diagnosis of postnatal depression. Our study interprets data from seven GPs based in one practice, and hence further research on a larger scale is required to confirm, refine or refute our results as well as uncover further possible configurations.

## Supplementary material

For supplementary material accompanying this paper visit https://doi.org/10.1017/S1463423620000316. click here to view supplementary material
