# Supplementary material for: Towards an understanding of GPs’ viewpoint on diagnosing postnatal depression in general practice: a small-scale realist evaluation
Source: Prim Health Care Res Dev. 2020 Oct 9;21:e42. doi: 10.1017/S1463423620000316 (PMC7576521; doi:10.1017/S1463423620000316)
Supplement: Supplementary file 1 [file S1463423620000316sup001.docx]

**Supplementary materials**

CASE SCENARIOS

**Case 1**

A 23-year-old woman who is 13-weeks postpartum has booked a check-up with you. She says that she’s had a stomach ache for the past couple of weeks and has seen a specialist about it but they were unable to find the cause of it. When you ask her how she’s coping with being a mother she says that she’s doing just fine but her appetite has reduced, she’s finding it difficult to sleep, she feels like she has less energy and is quite irritable due to her stomach ache. When you ask her about her mood she again says that she’s fine and that she just wants to find out how to treat her stomach ache because it’s really bothering her.

**Case 2**

A 31-year-old woman has a 8-week postnatal check-up booked with you and brings her husband along with her. Her husband explains that they are really enjoying having a new addition to the family. When you ask the woman about her mood her husband answers for her and says that she is loving being a mother, and the woman doesn’t make eye contact and seems quite tearful.

**Case 3**

A 19-year-old woman who is 12 weeks postpartum is due for a check-up with you. When you ask her about her relationship with her new baby she says that her and her baby are doing just fine.

When you ask her more questions you find out that she has been finding it difficult to sleep, her appetite has reduced, she has not seen her friends or family (other than her partner) for about 6 weeks, and that she hasn’t been able to enjoy any activities she used to do for fun such as reading, yoga and seeing her friends. However, she assures you that she’s fine and says that it’s normal to feel this way after you’ve just had a baby.

**Case 4**

A 26-year-old woman is due her 6-week postnatal check-up. She is gravida 2 and has a medical history of postnatal depression from her first pregnancy. She presents with low mood, lack of energy, and says that she is recently finding it much more difficult to get up in the morning. She states that she thinks she might be depressed again and wants your opinion.

**Appendix 1: Interview Schedule**

Case scenario questions

1. What initial thoughts do you have of this patient’s health and well-being?

- What aspects of this scenario make you think this?
- What aspects sound most reassuring/concerning?

1. What further questions might you ask this patient?

- What further information may you require?
- What aspects of this scenario would you want to explore further?

1. What would you do to proceed clinically?

- How would you diagnostically approach this patient case?
- Are there any other professionals you would involve or ask to help/guide you?

1. Why have you chosen this diagnostic approach?

- Is this an approach that has worked in the past or you’ve used generally?
- Do you have any experience with this that suggests this approach would be effective?
- Are there any aspects of this scenario that encouraged you to use this approach? (age, relationship status, number of children, amount of time in post-partum)
- Are there any reasons that this approach would be most suitable for this particular patient?
- Are there any patient circumstances in which you would not choose this diagnostic approach?

1. Has the patient’s and your awareness of their condition (or lack of) affected your diagnostic approach? How?

- Do you have any previous experience that has led to this chosen approach?

1. Do you have any further comments for this case scenario?

Repeat the schedule for each of the case scenarios.

General questions about diagnostic approaches to postnatal depression.

1. Research suggests that postnatal depression is underdiagnosed. Why do you think this might be?

- Do you think the diagnosing process is easy/difficult? Have you faced any challenges?
- Do any previous difficult diagnosing experiences for postnatal depression come to mind?
- What do you think would help increase diagnostic rates?

1. What diagnostic tools have you used for postnatal depression?

- Does it vary according to the patient (e.g. age, postpartum period, number of children, family status)?

1. Do you have any experience using postnatal depression screening questionnaires, or any knowledge about them?

(If they have no knowledge or experience on screening tools then show them a print-out of EPDS and PHQ-9.)

- What do you believe are their main strengths and weaknesses?
- In which contexts or patient scenarios would you consider or avoid using screening questionnaires? Why?

1. Do you think other members of the primary care team contribute to the diagnostic process? How? Examples?

- Whose responsibility is it to detect signs and symptoms, check patient histories, make an official diagnosis etc?
- What role do you believe you play in the diagnostic process compared to the rest of the primary care team?

1. Is there anything else you would like to mention about postnatal depression diagnosis?
2. Do you have any questions for me?

CLOSE
